# Metabolomic Analysis Provides New Insight Into Tolerance of Huanglongbing in Citrus

**DOI:** 10.3389/fpls.2021.710598

**Published:** 2021-08-04

**Authors:** Joon Hyuk Suh, Xixuan Tang, Yi Zhang, Frederick G. Gmitter, Yu Wang

**Affiliations:** Citrus Research and Education Center, University of Florida, Lake Alfred, FL, United States

**Keywords:** citrus, Huanglongbing (HLB), tolerance, plant growth, phloem regeneration, plant defense, metabolic pathway, metabolomics

## Abstract

There have been efforts to develop citrus cultivars that are tolerant of Huanglongbing (HLB), a catastrophic phloem-limited disease. Previous studies demonstrated that continuous plant growth with phloem regeneration is one of the major characteristics of HLB tolerance. In this study, the metabolic mechanisms of HLB tolerance in citrus were elucidated using a multiple pathway-targeted metabolomic approach. Comparative analysis of healthy and infected HLB-tolerant and HLB-sensitive mandarin cultivars (*Citrus reticulata*) revealed differentially expressed metabolic responses among different groups. Pathway enrichment analysis indicated aspartate and glutamate metabolism, purine metabolism, and biosynthesis of plant hormones were upregulated in the tolerant group, except salicylic acid signaling. Catabolic pathways linked to energy-yielding metabolism were also upregulated in the tolerant group. These metabolisms and pathways were interconnected with each other, unveiling a pivotal metabolic network associated with HLB tolerance. In the network, auxins and cytokinins, the plant hormones responsible for plant growth and phloem regeneration, were accumulated. In addition, purine metabolites serving as energy carriers and nitrogen sources of plants were increased. Only salicylic acid-related metabolites for plant defense responses were decreased in the tolerant group. Our findings may evidence the strategy of HLB-tolerant cultivars that sustain plant growth and phloem formation rather than displaying direct plant defense to overcome the disease.

## Introduction

Huanglongbing (HLB), also called citrus greening, is one of the most destructive diseases of citrus, presumably caused by phloem-limited bacteria named *Candidatus* Liberibacter asiaticus (*Ca*Las), which is transmitted to the host by Asian citrus psyllids (*Diaphorina citri*) (Jagoueix et al., 1994; Bové, 2006). HLB was first discovered in the areas of Asia and Africa and has spread to Brazil in 2004 and the USA in 2005 (Halbert, 2005; Texeira et al., 2005). HLB symptoms include leaf vein yellowing, foliar blotchy mottle, and asymmetrical chlorosis, followed by tree decline, leaf loss, and premature fruit drop. HLB has decimated millions of citrus trees, with a decrease in fruit quality and yield, resulting in a significant impact on global citrus production (Kramer et al., 2020; USDA, 2020). In the USA, HLB is prevalent in Florida where more than 95% of citrus groves are infected by *Ca*Las (Kramer et al., 2020). The production of oranges is now less than one-third of what it was 20 years ago, leading to severe economic losses to the citrus industry (USDA, 2020).

There are major challenges in HLB studies and management. First, there is no effective cure for HLB. Although several techniques with chemical application and management practices have been used, they could not control the disease (Pitino et al., 2020). In addition, *Ca*Las has not been successfully isolated and cultured *in vitro*, making it more difficult to characterize the disease and to fully understand pathogenesis mechanisms, such as a very early stage of host–microbe interaction (Zuñiga et al., 2020). These difficulties have led to alternative strategies for dealing with HLB, such as developing new cultivars with HLB tolerance or resistance. To date, no citrus is truly resistant to HLB. However, some citrus cultivars have shown greater tolerance than others under natural HLB conditions (Albrecht and Bowman, 2012; Wu et al., 2020). ‘LB8-9’ Sugar Belle^®^ mandarin hybrid (*C. reticulata* ‘Clementine’ mandarin × ‘Minneola’ tangelo) is one of the promising tolerant cultivars, with proven evidence that its growth and fruit yield are not substantially suppressed despite the presence of HLB symptoms (Gmitter et al., 2010; Stover et al., 2016).

At the anatomical level, citrus (e.g., leaf tissue) exhibits visible changes induced by *Ca*Las infection. The alterations include phloem necrosis, sieve element plugging, and phloem collapse, with cell wall thickening and distortion (Fan et al., 2013). Since the phloem is responsible for transporting photosynthates from the source to the sink organs, phloem disruption restricts the movement of nutrients, potentially leading to abnormal starch accumulation with the typical symptoms of HLB disease (Fan et al., 2010). The phloem dysfunction seems to be related to the inherent properties of *Ca*Las, exclusively residing in the sieve tubes of the phloem, and exploiting nutrients available in the phloem (Jagoueix et al., 1994; Hijaz et al., 2016a). Interestingly, the significant changes of phloem, such as phloem cell collapse, are found less in HLB-tolerant citrus species (Fan et al., 2013). This provides a clue on the link between phloem function/health and HLB tolerance. Recent studies demonstrated that tolerant cultivars (e.g., Sugar Belle^®^) tend not only to favorably preserve their internal plant structures but also to actively regenerate new phloem compared with sensitive selections (Deng et al., 2019). Rapid replacement of phloem could compensate for the old disrupted phloem caused by the infection. These results suggest that sustaining plant growth, with lower levels of phloem damage and greater phloem regeneration, is a key element that contributes to HLB tolerance (Fan et al., 2013; Deng et al., 2019).

Although phenotypic observations at the anatomical and physiological levels revealed the importance of continuous growth and phloem regeneration in HLB tolerance, the biological pathways and networks underlying these features have not been fully investigated. There were genomic and transcriptomic studies to identify gene candidates associated with HLB tolerance (Fan et al., 2012; Rawat et al., 2017; Shi et al., 2018; Wu et al., 2020), while only a few genes were found to support the mechanism of phloem regeneration (e.g., cell wall-related genes) (Fan et al., 2012; Wu et al., 2020). In the wake of genomics and transcriptomics, several metabolomic studies have been performed to identify HLB tolerance-specific metabolites, but their findings were limited to metabolomes with antimicrobial activities involved in direct plant defense, neglecting phloem physiology, and sustained plant growth in the HLB-tolerant hosts (Albrecht et al., 2016; Hijaz et al., 2016b; Killiny et al., 2017, 2018; Zou et al., 2019).

In this study, we used a multiple pathway-targeted metabolomic approach covering a broad range of primary and secondary metabolic pathways including carbohydrate metabolism (e.g., glycolysis/gluconeogenesis, citrate cycle, and metabolism of sugar), nucleotide metabolism (e.g., purine and pyrimidine), amino acid metabolism (e.g., alanine, aspartate, glutamate, glycine, serine, and threonine), energy metabolism (e.g., carbon fixation), and biosynthesis of secondary metabolites (e.g., flavonoid and plant hormone) to elucidate metabolic mechanisms regarding plant growth and phloem regeneration associated with HLB tolerance in citrus. The metabolic responses from HLB-tolerant (Sugar Belle^®^ mandarin) and HLB-sensitive (‘Murcott’ mandarin, *C. reticulata* Blanco) cultivars were compared and were further interpreted and integrated to identify disease tolerance markers and core metabolic pathways and networks.

## Materials and Methods

### Plant Materials

Sugar Belle^®^ mandarin (HLB-tolerant cultivar; *C. reticulata* ‘Clementine’ mandarin × ‘Minneola’ tangelo) and ‘Murcott’ mandarin (HLB-sensitive cultivar; *C. reticulata* Blanco) trees were grown in the greenhouse at the University of Florida's Citrus Research and Education Center (Lake Alfred, FL, USA). The budwood of Sugar Belle^®^ and ‘Murcott’ were grafted onto sour orange (*Citrus aurantium* L.) rootstock. One year after grafting, the seedlings were graft-inoculated with budwood from pathogen-free and *Ca*Las-infected sour orange to generate healthy and infected trees, respectively. For all trees, the presence of *Ca*Las was confirmed by quantitative real-time polymerase chain reaction (qPCR) analysis (Li et al., 2006). One year later, 5–7 leaf samples were randomly collected from three individual trees (biological replicate, *n* = 3) of healthy Sugar Belle^®^ (healthy HLB-tolerant group), infected Sugar Belle^®^ (infected HLB-tolerant group), healthy ‘Murcott’ (healthy HLB-sensitive group), and infected ‘Murcott’ (infected HLB-sensitive group) (Table 1). The collected samples were immediately frozen in liquid nitrogen (N_2_) and kept at −80°C until use.

**Table 1:** General information of HLB-tolerant and HLB-sensitive cultivars used in this study.

**Cultivar**	**Rootstock**	**Sample code**	**Ct value^a^**	**Status**	**HLB sensitivity**
Sugar Belle^®^ mandarin	Sour orange	H/SB-1	31.79	Healthy	Tolerant
		H/SB-2	33.63	Healthy	Tolerant
		H/SB-3	34.41	Healthy	Tolerant
		I/SB-1	20.11	Infected (HLB)	Tolerant
		I/SB-2	20.01	Infected (HLB)	Tolerant
		I/SB-3	20.05	Infected (HLB)	Tolerant
‘Murcott’ mandarin	Sour orange	H/M-1	34.16	Healthy	Sensitive
		H/M-2	35.68	Healthy	Sensitive
		H/M-3	35.15	Healthy	Sensitive
		I/M-1	22.15	Infected (HLB)	Sensitive
		I/M-2	20.22	Infected (HLB)	Sensitive
		I/M-3	18.95	Infected (HLB)	Sensitive

a *Ct value < 30 (HLB-positive)*.

### Sample Preparation

The collected leaf samples were placed into a stainless-steel grinding jar set and precooled in liquid nitrogen (N_2_) (−80°C) for 2–5 min. After freezing the grinding jars, the samples were disrupted and homogenized using stainless-steel grinding balls with a TissueLyser at 20–30 Hz for 2 min. For the reversed-phase (RP) chromatographic analysis, 50 mg of samples were mixed with 1 ml of 20% methanol containing internal standard mixtures (citric-2,2,4,4-d_4_ acid, hippuric acid-d_5_, salicylic acid-d_6_, and apigenin-d_5_). For hydrophilic interaction chromatographic (HILIC) analysis, the samples were mixed with 1 ml of 90% methanol containing internal standard mixtures (l-aspartic acid-2,3,3-d_3_, d-fructose-^13^C_6_, d-sorbitol-^13^C_6_, and guanine-4,5-^13^C2,7-^15^N). The samples were vortexed for 10 min, followed by sonication for 30 min in an ice block-filled bathtub. After centrifugation at 30,000 g at 4°C for 10 min, the supernatant was filtered using 0.22-μm nylon syringe filters (Bonna-Agela Technologies Inc., Wilmington, NC, USA) and was injected into the liquid chromatography–tandem mass spectrometry (LC–MS/MS) system. Quality control (QC) samples were prepared by mixing aliquots of all samples with the same sample preparation. Three technical replicates (*n* = 3) were performed for each sample (three biological replicates and three technical replicates, total *n* = 9).

### Multiple Pathway-Targeted Metabolomics

For the multiple pathway-targeted metabolomics, all metabolites were thoroughly confirmed using authentic standards. For the evaluation of data quality, pooled QC samples were injected after every nine samples and assessed throughout the analysis. A mass spectrometer (TSQ Quantiva, Thermo Fisher Scientific, San Jose, CA, USA) was equipped with an electrospray ionization (ESI) interface and operated in both positive and negative ionization modes depending on the analytes. Selective reaction monitoring was used for metabolite detection. For the RP LC–MS/MS analysis, analytes were eluted on the Thermo Acclaim C30 column (2.1 × 150 mm, 3 μm particle size) at a column temperature of 25°C using (A) 0.1% of formic acid in water and (B) 0.1% of formic acid in acetonitrile as mobile phases. A gradient elution at a flow rate of 0.2 ml/min was carried out as follows: 0–3 min, 2% B; 3–25 min, 2–50% B; 25–30 min, 50–90% B; 30–30.1 min, 90–95% B; 30.1–35 min, 95% B; 35–35.1 min, 95–2% B; and 35.1–45 min, 2% B. The ESI parameters were as follows: positive spray voltage, 3,500 V; negative spray voltage, 2,500 V; ion transfer tube temperature, 325°C; vaporizer temperature, 275°C; sheath gas, 35 Arb; aux gas, 10 Arb; and sweep gas, 0 Arb. MS/MS parameters were optimized for each metabolite using the flow injection analysis of individual standards. Collision-induced dissociation (CID) gas pressure was set at 2 mTorr with a dwell time of 50 ms. The optimum values are listed in Supplementary Table 1. The group of plant hormones, organic acids, and flavonoids were analyzed under the RP mode (Supplementary Table 1).

For HILIC LC–MS/MS analysis, two HILIC columns (HILIC 1 and 2) were employed to detect sugars, organic acids, amino acids, nucleotides, nucleosides, and others. HILIC 1 was an Agilent Poroshell 120 HILIC-Z (2.1 × 150 mm, 2.7 μm particle size) column, and HILIC 2 was a Merck SeQuant ZIC-p HILIC (2.1 × 100 mm, 5.0 μm particle size) column. The detailed information of metabolites and HILIC columns are shown in Supplementary Table 1. The HILIC 1 column was used with mobile phases of (A) 10 mM ammonium acetate in water (pH 9.0) and (B) 10 mM ammonium acetate in water–acetonitrile (10:90, v/v, pH 9.0). The gradient elution was carried out as follows: 0–2 min, 90% B; 2–15 min, 90–51% B; 15–20 min, 51% B; 20–21 min, 51–90% B; and 21–30 min, 90% B. The column temperature was 30°C, and the flow rate was at 0.25 ml/min. The mass spectrometer parameters were controlled as follows: positive spray voltage, 3,500 V; negative spray voltage, 2,500 V; ion transfer tube temperature, 325°C; vaporizer temperature, 290°C; sheath gas, 38 Arb; aux gas,11 Arb; and sweep gas, 0 Arb. The HILIC 2 column was used with mobile phases of (A) 10 mM ammonium acetate with 0.25 mM methylphosphonic acid in water (pH 9.0) and (B) 10 mM ammonium acetate in water–acetonitrile (10:90, v/v, pH 9.0). The gradient elution was selected as follows: 0–4 min, 95–40% B; 4–11 min, 40–35% B; 11–13 min, 35–95% B; and 13–24 min, 95% B. The flow rate was 0.15 ml/min, and the column temperature was 40°C. The mass parameters were as follows: positive spray voltage, 3,500 V; negative spray voltage, 2,500 V; ion transfer tube temperature, 325°C; vaporizer temperature, 210°C; sheath gas, 27 Arb; aux gas, 9 Arb; and sweep gas, 0 Arb. Optimum MS/MS parameters for both HILIC 1 and 2 are presented in Supplementary Table 1. Both modes employed CID gas pressure at 2 mTorr with a dwell time of 25 ms.

### Data Processing and Statistics

Data processing of the pathway-targeted metabolomics was performed using Xcalibur version 3.0 software (Thermo Fisher Scientific, San Jose, CA, USA). Metabolic responses were calculated and normalized by the peak area ratios of analytes to internal standards. Multiple isotope-labeled internal standards were used for the normalization of the data (Supplementary Table 1). Analytes with relative standard deviation (RSD) values of QC samples higher than 30% (QC RSD value > 30%) were excluded from data interpretation and statistical analyses. The principal component analysis (PCA) and partial least squares discriminant analysis (PLS-DA) were conducted using SIMCA-P+ version 15.0 (Umetrics, Umeå, Sweden) to assess metabolic differences between groups. The variable importance in projection (VIP) scores acquired from PLS-DA results. SPSS software version 20.0 (IBM, Armonk, NY, USA) was used for the unpaired *t*-test. The *p*-values (<0.05) from the *t*-test, VIP scores (>1.0), and absolute fold changes (>1.5) were employed as criteria for selecting marker compounds. The hierarchical clustering heat maps were generated based on Pearson's distance measure and Ward's clustering algorithm with log-transformed and mean-centered data using MetaboAnalyst version 4.0 (http://metaboanalyst.ca) (Xia et al., 2009). The metabolic pathway enrichment analysis used MBRole version 2.0 (http://csbg.cnb.csic.es/mbrole2) (Chagoyen and Pazos, 2011; López-Ibáñez et al., 2016) with the Kyoto Encyclopedia of Genes and Genomes (KEGG, http://genome.jp/kegg) (Kanehisa and Goto, 2000) pathway database. Based on the enrichment results, the pathway mapping was performed using VANTED version 2.6.3 (http://vanted.ipk-gatersleben.de) with its SBGN-ED add-on (Junker et al., 2006).

## Results

### Phenotypic Changes in HLB-Tolerant and HLB-Sensitive Cultivars After Infection

Healthy and *Ca*Las-infected Sugar Belle^®^ mandarin (HLB-tolerant group) and ‘Murcott’ mandarin (HLB-sensitive group) trees were selected from the greenhouse at the University of Florida's Citrus Research and Education Center (Lake Alfred, FL, USA). The general information of plant materials used in this study is described in Table 1. *Ca*Las infection was confirmed by using the qPCR analysis with *Ca*Las-specific primers (Li et al., 2006). The infected trees were PCR positive with cycle threshold values under 30, which are confidently considered “infected” (Table 1) (Lee et al., 2015). When leaf samples were collected for the metabolomic study (1 year after graft-inoculation with pathogen-free and *Ca*Las-infected budwoods), infected ‘Murcott’ exhibited subtle phenotypic changes, such as downward curvature of leaves and mild thickening, while neither the infected ‘Murcott’ nor the infected Sugar Belle^®^ showed major HLB symptoms (e.g., asymmetric blotchy mottles and yellow veins). By observing them one more year after sample collection, both were found to undergo phenotypic alterations, but symptoms were more severe in the infected ‘Murcott’ than in the infected Sugar Belle^®^ (Supplementary Figure 1). Some infected ‘Murcott’ trees declined with leaf loss, which did not happen to the infected Sugar Belle^®^ samples. Healthy Sugar Belle^®^ and ‘Murcott’ trees had no signs of pathological symptoms in 2 years after graft inoculation.

### Metabolic Variations in HLB-Tolerant and HLB-Sensitive Cultivars

Metabolite profiles were examined in healthy and infected Sugar Belle^®^ (HLB-tolerant group) and ‘Murcott’ (HLB-sensitive group). A total of 178 metabolites, including 16 sugars, 21 amino acids, 22 organic acids, 21 nucleosides, 32 nucleotides, 29 flavonoids, 31 plant hormones, and 6 others were analyzed using LC–MS/MS (Supplementary Table 1). These metabolites covered a broad spectrum of primary and secondary metabolic pathways including carbohydrate metabolism (e.g., glycolysis/gluconeogenesis, citrate cycle, and metabolism of sugar), nucleotide metabolism (e.g., purine and pyrimidine), amino acid metabolism (e.g., alanine, aspartate, glutamate, glycine, serine, and threonine), energy metabolism (e.g., carbon fixation), and biosynthesis of secondary metabolites (e.g., flavonoid and plant hormone). Three biological replicates and three technical replicates (*n* = 9) were performed for each group (total of four groups, namely, healthy HLB-tolerant group, healthy HLB-sensitive group, infected HLB-tolerant group, and infected HLB-sensitive group). All analytes were confirmed using authentic standards by comparing their retention times and selected precursor and production pairs.

**Figure 1:**
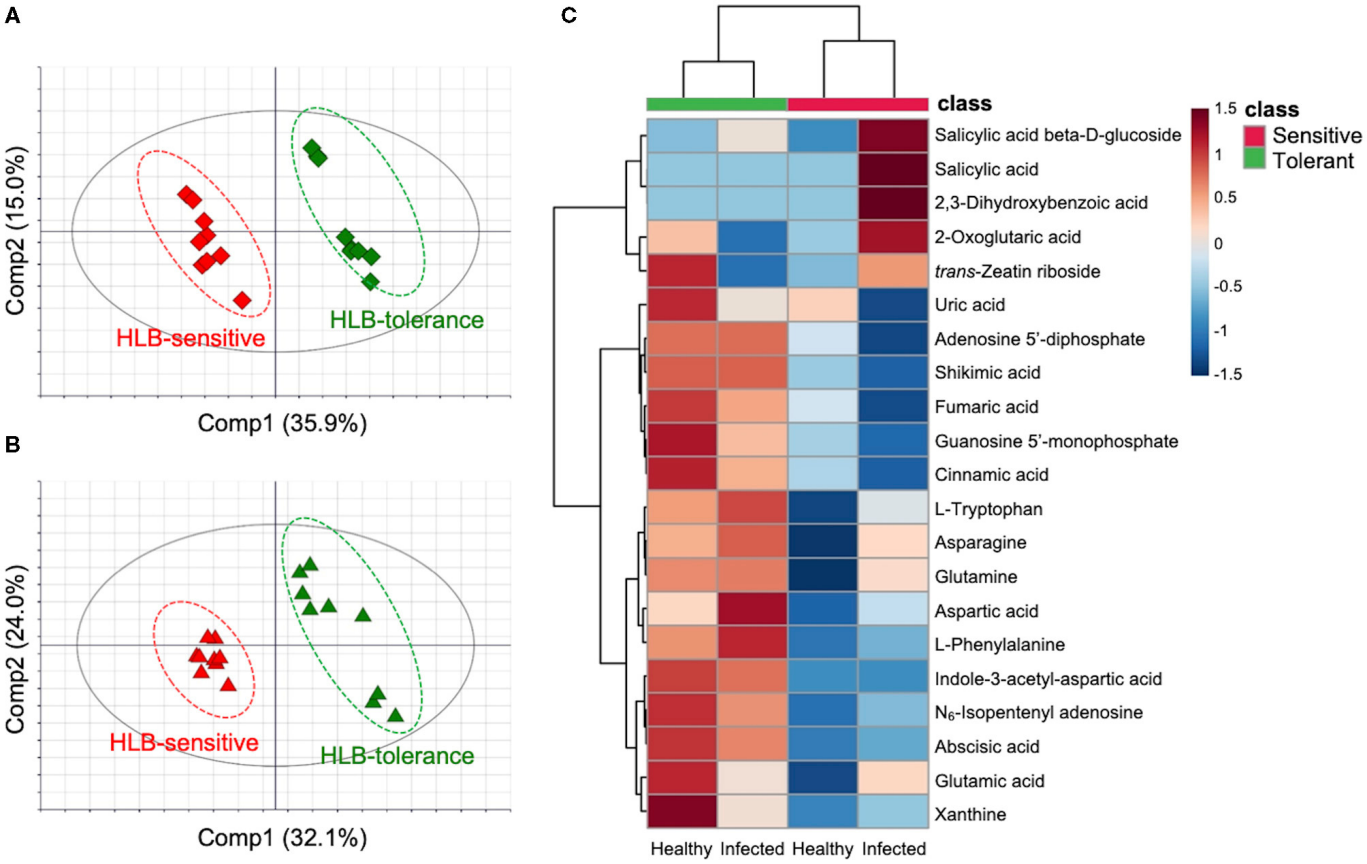
Metabolic differences between Huanglongbing (HLB)-tolerant (Sugar Belle^®^) and HLB-sensitive (‘Murcott’) citrus cultivars (*n* = 9). Partial least squares discriminant analysis score scatter plots of **(A)** healthy and **(B)** infected groups including HLB-tolerant samples (green) and HLB-sensitive samples (red). **(C)** Hierarchical clustering heat map of metabolites in four groups (healthy and infected HLB-tolerant and HLB-sensitive samples). All visualized metabolites are marker compounds [Variable importance in projection (VIP) score > 1.0, *p*-value < 0.05, and |fold change| > 1.5] involved in the pivotal pathways biosynthesis of plant hormones (BPH), aspartate and glutamate metabolism (AGM), and purine metabolism (PM)] related to HLB tolerance. The color depth of the heat map represents the degree of metabolic responses as follows: red color denotes increased responses and blue color denotes decreased responses.

First, the healthy HLB-tolerant group was compared with the healthy HLB-sensitive group (healthy group comparison). PCA clearly distinguished two groups, explained by 36.5% of the total variation on the PC1 axis (Supplementary Figure 2A), revealing discriminative metabolic responses between groups. PLS-DA was used to evaluate the VIP scores of metabolites. The PLS-DA model was thoroughly validated by a 999 × permutation test and ANOVA of the cross-validated residuals (CV-ANOVA) test (Eriksson et al., 2008). An obtained PLS-DA score scatter plot is shown in Figure 1A. Pivotal metabolites potentially related to the disease tolerance were identified by three criteria including multivariate statistical parameters (VIP scores), univariate statistical parameters (*p*-values), and fold changes. Of the 178 metabolites, only those with a VIP score over 1.0 (VIP score > 1.0), a *p*-value below 0.05 (*p*-value < 0.05), and an absolute fold change larger than 1.5 (|fold change| > 1.5) were considered as marker compounds possibly associated with the disease responses. Metabolites selected from the comparison on healthy groups are shown in Supplementary Table 2. Then, the metabolic differences were further confirmed by using infected HLB-tolerant and infected HLB-sensitive groups (infected group comparison). PCA again classified the samples into “HLB-tolerant” and “HLB-sensitive,” explained by 58.3% on PC1 and PC2 axes (Supplementary Figure 2B). The subsequent PLS-DA results are shown in Figure 1B. Based on the three criteria described earlier, metabolite markers were also obtained from the infected group comparison (Supplementary Table 3).

Integrated marker compounds from healthy and infected group comparisons are shown in Supplementary Figure 3. A total of 50 metabolites were considered as potential markers related to the tolerance. Four types of metabolic behaviors were observed as follows: (1) metabolites always increased in the HLB-tolerant group regardless of healthy/infected status, (2) metabolites always decreased in the HLB-tolerant group regardless of healthy/infected status, (3) metabolites increased in the HLB-tolerant group under the healthy condition while decreased under the infection, and (4) metabolites decreased in the HLB-tolerant group under the healthy condition while increased under the infection. Of these trends, more than 80% of metabolites (43 out of 50) were found to follow behaviors 1 and 2 (constant behaviors). Notably, most of the metabolites with constant behaviors (33 out of 43) acted as behavior 1 (metabolites always increased in the tolerant group), while only a few (10 out of 43) acted as behavior 2 (metabolites always decreased in the tolerant group). The metabolite markers with behaviors 1 and 2 are shown in a heat map derived from the hierarchical clustering analysis (Supplementary Figure 4). The clustering analysis first separated samples into two major clusters (HLB-tolerant and HLB-sensitive groups) according to the disease tolerance and separated each cluster again into two subclusters (healthy and infected groups) according to the disease status. This revealed that the metabolic variations were predominantly linked to cultivars with different levels of tolerance to disease, not the disease itself, although metabolic changes by the disease gave some clues on whether the metabolites are truly correlated with the tolerance or not.

### Metabolic Pathway Analysis Related to HLB Tolerance

**Figure 2:**
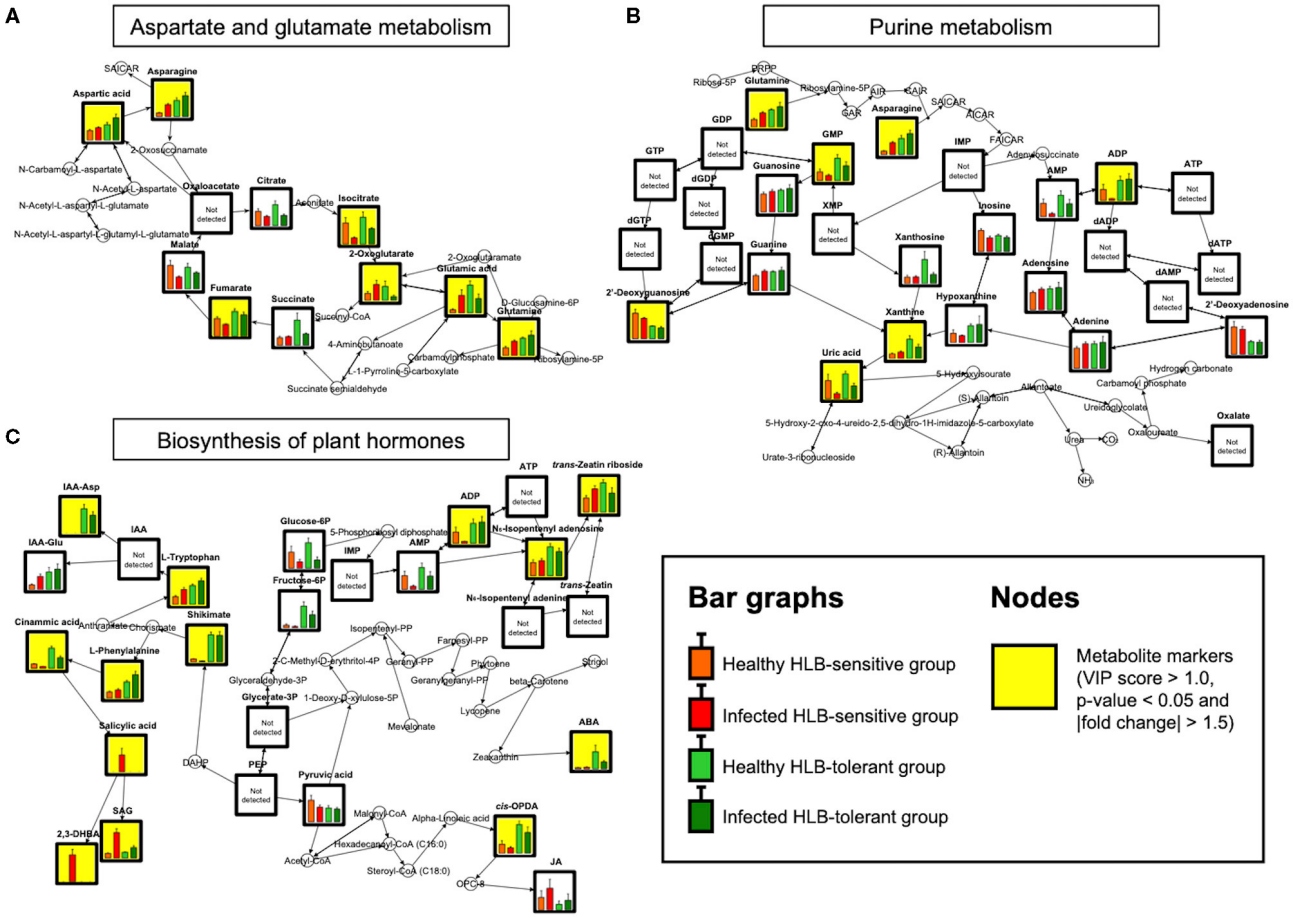
Metabolite mapping on **(A)** AGM **(B)** PM, and **(C)** BPH pathways (*n* = 9). Bar graphs show metabolite levels with SE in healthy HLB-sensitive group (orange), infected HLB-sensitive group (red), healthy HLB-tolerant group (light green), and infected HLB-tolerant group (green) based on the integrated results. Yellow background indicates marker compounds (VIP score > 1.0, *p*-value < 0.05, and |fold change| > 1.5) in the pathways.

To identify relationships between metabolites and biological pathways, pathway enrichment analysis was performed using the aforementioned 50 marker compounds. Among the metabolites, 36 compounds were recognized in the KEGG database (Kanehisa and Goto, 2000). Pathways showing high matched/total metabolites, with low adjusted *p*-values and false discovery rate values (<0.05), were considered as candidate pathways potentially related to HLB tolerance. Three metabolic pathways met the criteria and had a high correlation with the metabolite markers, namely, biosynthesis of plant hormones (BPH), aspartate and glutamate metabolism (AGM), and purine metabolism (PM) (Table 2). A heat map was constructed on the metabolites in these pathways (BPH, AGM, and PM) from the four groups (healthy HLB-tolerant group, infected HLB-tolerant group, healthy HLB-sensitive group, and infected HLB-sensitive group). As seen in Figure 1C, most of the metabolites in the pathways were shown to be increased in the tolerant group (behavior 1) regardless of healthy/infected status. Only salicylic acid-related metabolites (e.g., salicylic acid, salicylic acid β-d-glucoside, and 2,3-dihydroxybenzoic acid) were decreased in the tolerant group. Subsequently, metabolite mapping on the candidate pathways (BPH, AGM, and PM) was conducted using integrated metabolic response data from the four groups (i.e., healthy HLB-tolerant group, infected HLB-tolerant group, healthy HLB-sensitive group, and infected HLB-sensitive group) (Figure 2). The mapping results indicated that the pathways were overall upregulated in the tolerant group, except salicylic acid signaling.

**Table 2:** Results of metabolic pathway enrichment analysis.

**Pathway**	**Matched/ total metabolites**	***P*-value**	**FDR correction**
Biosynthesis of plant hormones	12/36	5.18e^−11^	5.28e^−09^
Aspartate and glutamate metabolism	6/36	1.91e^−08^	4.37e^−07^
Purine metabolism	6/36	6.87e^−05^	5.39e^−04^

### Metabolic Network Involved in HLB Tolerance

**Figure 3:**
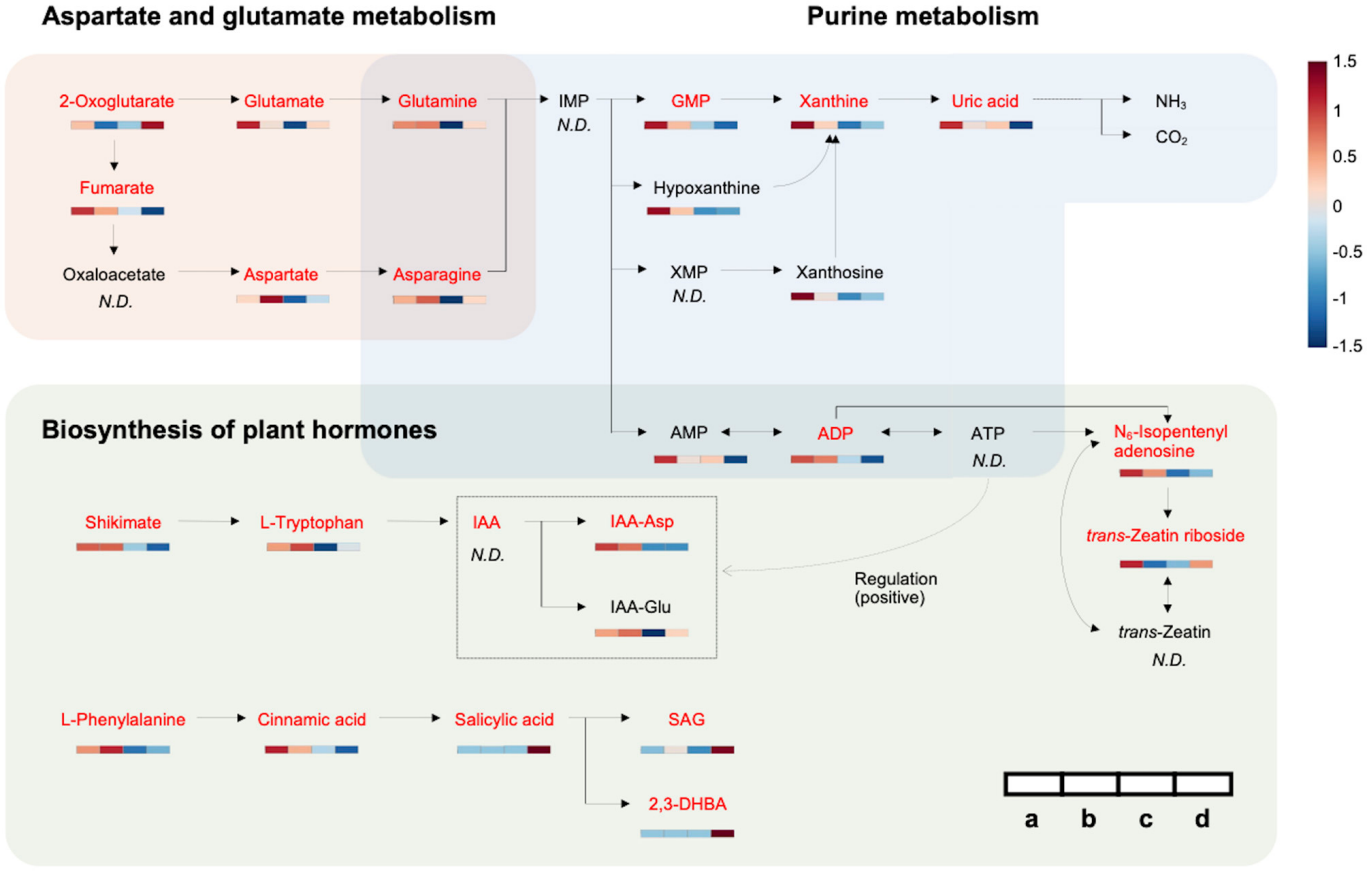
Metabolic network associated with HLB tolerance based on an integrated pathway. Heat maps show metabolic variations in healthy and infected HLB-tolerant and HLB-sensitive groups [(a) healthy HLB-tolerant group, (b) infected HLB-tolerant group, (c) healthy HLB-sensitive group, and (d) infected HLB-sensitive group]. Metabolites with red color indicate marker compounds (VIP score > 1.0, *p*-value < 0.05, and |fold change| > 1.5) in the network. The color depth of the heat map represents the degree of metabolic responses as follows: red color denotes increased responses and blue color denotes decreased responses.

**Figure 4:**
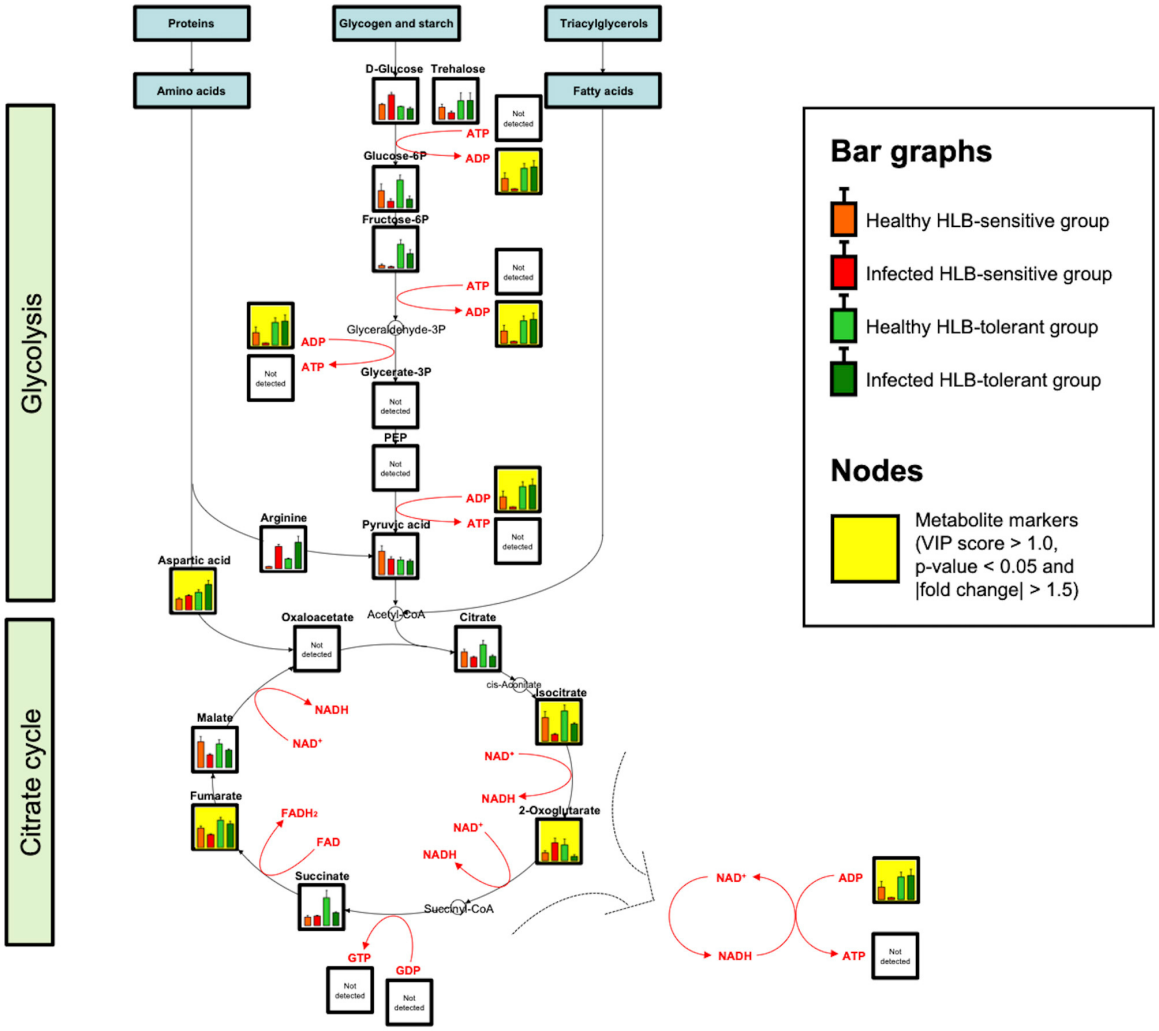
Catabolic pathways and metabolite mapping (*n* = 9). Central catabolic pathways including glycolysis and citrate cycle are investigated and visualized. Bar graphs show metabolite levels with SE in healthy HLB-sensitive group (orange), infected HLB-sensitive group (red), healthy HLB-tolerant group (light green), and infected HLB-tolerant group (green) based on the integrated results. Yellow background indicates marker compounds (VIP score > 1.0, *p*-value < 0.05, and |fold change| > 1.5) in the pathways.

A metabolic network related to HLB tolerance was identified based on the results of metabolic pathway analysis and metabolite mapping. The candidate pathways (BPH, AGM, and PM) were found to be interconnected with each other. Among them, the PM pathway was revealed to be a core metabolic pathway connected to the other two pathways (BPH and AGM). The downstream metabolites (asparagine and glutamine) of the AGM pathway are used as the upstream substrates of the PM pathway. The intermediate metabolites of the PM pathway, such as adenosine 5′-monophosphate (AMP), adenosine 5′-diphosphate (ADP), adenosine 5′-triphosphate (ATP), guanosine 5′-monophosphate (GMP), xanthine, and uric acid, are synthesized from these substrates (asparagine and glutamine). Some of the derived metabolites, such as AMP, ADP, and ATP, are further used as substrates for cytokinins (e.g., N_6_-isopentenyl adenosine, *trans*-zeatin riboside, and *trans*-zeatin) of the BPH pathway. ATP is also used for an efflux transporter (ATP-binding cassette subfamily B) regulating the level of auxins [e.g., indole-3-acetic acid (IAA), indole-3-acetyl-aspartic acid (IAA-Asp), and indole-3-acetyl-glutamic acid (IAA-Glu)] in the BPH pathway (Sharma and Zheng, 2019). An integrated pathway (metabolic network) based on the above results is shown in Figure 3. In addition to the integrated pathway, we also investigated catabolic pathways linked to energy-yielding metabolism. Through the catabolic pathways (central pathways: glycolysis and citrate cycle), complex molecules (e.g., carbohydrates, proteins, and fats) are degraded into simpler ones, finally producing ATP and guanosine 5′-triphosphate (GTP), which are energy carriers used for various cellular processes (Sweetlove and Fernie, 2005; Gardeström and Igamberdiev, 2016). Interestingly, some metabolites (e.g., ADP, aspartate, fructose-6-phosphate, isocitrate, and fumarate) were found to be increased in the tolerant group within the catabolic pathways (Figure 4).

## Discussion

The observed differences in HLB symptoms between HLB-tolerant (Sugar Belle^®^) and HLB-sensitive (‘Murcott’) cultivars implied that there might be metabolic variations leading to different levels of tolerance. Mild HLB symptoms seen in the HLB-tolerant group were previously proven at the anatomical level, with phenomena including sustained plant growth and increased phloem regeneration (Fan et al., 2013; Deng et al., 2019). However, metabolic mechanisms underlying these features remain unknown. Therefore, in this study, a multiple pathway-targeted metabolomic approach was performed to identify pivotal metabolic pathways and networks related to HLB tolerance.

The unsupervised PCA results revealed discriminative metabolic responses in the HLB-tolerant and HLB-sensitive groups (Supplementary Figure 2). Following PLS-DA (supervised), results showed further separation between groups based on the metabolic differences (Figures 1A,B). By cross-comparing healthy and infected groups (total four groups: healthy HLB-tolerant group, healthy HLB-sensitive group, infected HLB-tolerant group, and infected HLB-sensitive group), we could determine not only metabolite markers linked to the disease tolerance (Supplementary Figure 3) but also their distinctive behaviors in different groups by the infection (Supplementary Figure 4). Among the four types of observed behaviors (behavior 1: metabolites always increased in the tolerant group, behavior 2: metabolites always decreased in the tolerant group, behavior 3: metabolites increased in the tolerant group under the healthy condition while decreased under the infection, and behavior 4: metabolites decreased in the tolerant group under the healthy condition while increased under the infection), more than 80% of metabolites followed behaviors 1 and 2 (constant behaviors). The list of metabolites with different behaviors (behaviors 1–4) is shown in Supplementary Figure 3. Of these metabolites, metabolites with behavior 1 (metabolites always increased in the tolerant group) were dominant (Supplementary Figure 4), indicating HLB-tolerant hosts inherently accumulate these metabolites.

By investigating metabolic pathways and networks, three candidate pathways (BPH, AGM, and PM) and their constituent metabolites related to the tolerance were identified (Figure 1C, Table 2). Importantly, correlations between these pathways and HLB tolerance were reported for the first time. Among the pathways, the PM pathway was revealed as a core metabolic pathway connected to the other two pathways (BPH and AGM). The metabolites of the PM pathway are derived from the downstream metabolites of the AGM pathway, and some intermediate metabolites of PM pathways are used as substrates (or regulators) for plant hormones (auxins and cytokinins) of the BPH pathway. An integrated pathway clearly showed that metabolism along the candidate pathways (BPH, AGM, and PM) was upregulated in the tolerant group, except for salicylic acid signaling (Figure 3).

We found that the upregulated metabolism starts from two amino acids, namely, asparagine and glutamine, which are the downstream metabolites of the AGM pathway (Figure 2A). They were always increased in the tolerant group regardless of healthy/infected status. Remarkably, the levels of these amino acids were elevated in response to *Ca*Las infection in both HLB-tolerant and HLB-sensitive groups, implying that they possess some functions associated with host responses to the disease (and possibly disease tolerance). Several studies have reported increased amounts of these amino acids (asparagine and/or glutamine) under HLB disease or in the tolerant citrus cultivars (Hung and Wang, 2018; Killiny et al., 2018; Albrecht et al., 2020). However, they failed to elucidate metabolic mechanisms of why these metabolites are accumulated in the hosts. Our results demonstrated that the increased amino acids (asparagine and glutamine) dominantly serve as the upstream substrates of the PM pathway, leading to active generation (accumulation) of downstream metabolites in the pathway. In fact, in the tolerant group, elevated levels of intermediate metabolites related to *de novo* purine biosynthesis (e.g., AMP, ADP, and GMP) and purine degradation metabolism (e.g., xanthine and uric acid) in the PM pathway were observed (Figure 2B) (Ashihara et al., 2018). The accumulated *de novo* purine biosynthesis products, such as AMP and ADP, were proven to be further used for synthesizing cytokinins and regulating auxins in the BPH pathway. Increased amounts of cytokinins (e.g., N_6_-isopentenyl adenosine and *trans*-zeatin riboside) and auxins (e.g., IAA-Asp and IAA-Glu) in the tolerant group as shown in Figure 2C support the positive internal relationship of metabolites in AGM, PM, and BPH pathway cascades, except salicylic acid signaling (Figure 3).

Increased levels of purines such as AMP, ADP, and GMP in the tolerant group might also contribute to energy-yielding metabolism (catabolic pathways). ATP and GTP are the end products of this metabolism, and they require ADP and guanosine 5′-diphosphate (GDP) for their formation (Gardeström and Igamberdiev, 2016). ADP and GDP are derived from precursors, AMP and GMP, respectively. Thus, elevated AMP, ADP, and GMP can accelerate catabolism to generate more ATP and GTP. In addition to the accumulated purines (AMP, ADP, and GMP), some metabolites in the catabolic pathways were observed to be increased in the tolerant group as well (Figure 4). Although ATP and GTP were not detected in this study due to their low sensitivity, the elevated metabolic responses through these pathways indicated catabolism is possibly upregulated in HLB-tolerant cultivars.

Accumulation of metabolites might be involved in intrinsic mechanisms of HLB tolerance in the hosts. Auxins and cytokinins, the end products of the pathway cascades, are plant hormones that serve a key function in regulating plant growth and vascular tissue development (Chen et al., 2019; Sharma and Zheng, 2019). Auxins play a role in the regeneration and reunion of phloem tissues in the host plants (Chen et al., 2019). Cytokinins alone cannot fully regulate these processes, but along with auxins, they promote vascular differentiation and increase the phloem–xylem ratio (Aloni et al., 2006; Sharma and Zheng, 2019). The role of auxins and cytokinins in vascular tissue regeneration was previously confirmed through exogenous application of these hormones *in vitro* system (Chen et al., 2019). Another study also found that the treatment of auxins significantly increases citrus fruit growth, with the promotion of vascular tissue (xylem and phloem) development (Mesejo et al., 2003). Thus, the elevated levels of auxins and cytokinins in the tolerant group can lead to continuous plant growth and greater phloem regeneration, which were proven as the major phenotypic characteristics of HLB-tolerant cultivars (Fan et al., 2013; Deng et al., 2019). Notably, one of the auxins (IAA-Asp) was only found in HLB-tolerant cultivar, strongly supporting these features (Figure 2C). The accumulation of auxins and cytokinins seems to be caused by upregulated PM (*de novo* purine biosynthesis) and catabolic pathways because both pathways can actively generate ATP for producing and regulating these hormones (Figures 2B, 4). Especially, ATP and GTP derived from the catabolic pathways would be used for various cellular processes including the promotion of plant growth (Figure 4). However, the influence of upregulated catabolic pathways needs to be further investigated in the HLB-tolerant cultivars with follow-up studies. Meanwhile, another purine metabolite group (purine degradation metabolites), such as xanthine and uric acid, may also contribute to sustained plant growth because they are known to serve as nitrogen sources in plants by purine degradation processes to become NH_3_ (available form to plants) (Zrenner et al., 2006; Brychkova et al., 2008). It was reported that *Ca*Las infection causes nitrogen deficiency in citrus hosts (Mann et al., 2012). Thus, increased levels of the purine degradation metabolites in the tolerant group could help to prevent nitrogen deficiency in the infected hosts by a constant supply of nitrogen. In addition, an increased amount of xanthine may also be involved in the resistance mechanisms of the host against pathogen attack, which was confirmed by a metabolomic study using endophytes for biocontrol of *Ca*Las in citrus (Munir et al., 2020).

**Figure 5:**
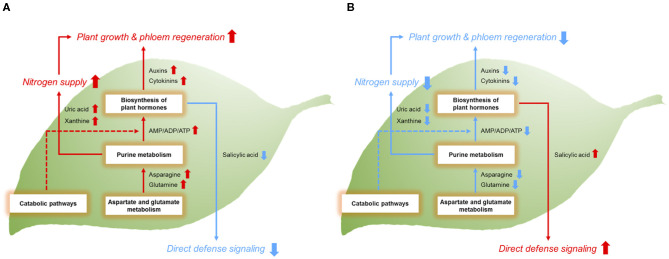
Metabolic mechanisms of **(A)** HLB-tolerant and **(B)** HLB-sensitive citrus hosts and physiological consequences. Red arrows represent the relative upregulation of metabolic responses or functional activities. Blue arrows represent the relative downregulation of these features. The dotted line indicates the metabolic pathway that needs to be further confirmed.

Although overall metabolic pathways were upregulated in the network, salicylic acid-related metabolites were conversely reduced in the tolerant group. Salicylic acid is responsible for plant defense mechanisms against a wide range of biotic stresses (Bari and Jones, 2009). Increased salicylic acid by *Ca*Las infection in the HLB-sensitive cultivars was observed not only in this study but also in a previous study (Nehela et al., 2018). However, diminished salicylic acid signaling in the HLB-tolerant cultivar was newly discovered in this study. It is not clear why salicylic acid responses were muted, but a recent study indicated that salicylic acid signaling induced by *Ca*Las infection is not always beneficial, and it sometimes occurs in the healthy tissues of infected trees, which are not yet affected by HLB (Martinelli and Dandekar, 2017). Therefore, decreased salicylic acid responses might be a part of tolerance strategies of HLB-tolerant hosts to favorably survive HLB. Taken together, our results suggest that HLB-tolerant cultivars may prefer continuous growth with new phloem formation direct activation of plant defense responses for the management of the disease. The upregulated metabolic pathways of AGM (e.g., asparagine and glutamine), PM (e.g., AMP, ADP, xanthine, and uric acid), and BPH (auxins and cytokinins) are thought to be involved in the sustained growth and phloem regeneration, while the downregulated salicylic acid signaling is likely to result in decreased defense responses in HLB-tolerant citrus trees. The opposite mechanisms seem to occur in HLB-sensitive citrus trees. We recently found evidence of gene expression to potentially support the phloem regeneration of the HLB-tolerant cultivar (Sugar Belle^®^, same trees used in this study) although it was not directly related to auxins and cytokinins (unpublished data). Follow-up studies will be needed to confirm our results at the molecular level. The metabolic mechanisms of the HLB-tolerant and HLB-sensitive hosts demonstrated in this study and physiological consequences are shown in Figure 5.

In this study, metabolic mechanisms underlying the major phenotypic traits of HLB-tolerant cultivars were identified using a multiple pathway-targeted metabolomic approach. We found a novel metabolic network linking upregulated pathway cascades with plant growth and phloem regeneration related to the disease tolerance. The role of key metabolites in the pathways revealed why overall metabolic responses in the network are positively correlated with the phenotypic features of the tolerance. Our findings provide a new perspective and framework for understanding the complex physiological mechanisms of HLB tolerance at the metabolite level. Furthermore, knowledge gained in this study may offer a clue on selecting markers related to the tolerance for future citrus breeding.

## Data Availability Statement

The original contributions presented in the study are included in the article/Supplementary Material, further inquiries can be directed to the corresponding author/s.

## Author Contributions

YW, FG, and JS conceptualized this study and wrote the manuscript. JS set up the metabolomic analytical platform and interpreted all experimental results. JS and XT performed metabolomic and statistical analyses. YZ conducted plant experiments, including qPCR analysis. All authors contributed to the article and approved the submitted version.

## Conflict of Interest

The authors declare that the research was conducted in the absence of any commercial or financial relationships that could be construed as a potential conflict of interest.

## Publisher's Note

All claims expressed in this article are solely those of the authors and do not necessarily represent those of their affiliated organizations, or those of the publisher, the editors and the reviewers. Any product that may be evaluated in this article, or claim that may be made by its manufacturer, is not guaranteed or endorsed by the publisher.
