# Supplementary material for: Metabolomic Analysis Provides New Insight Into Tolerance of Huanglongbing in Citrus
Source: Front Plant Sci. 2021 Aug 4;12:710598. doi: 10.3389/fpls.2021.710598 (PMC8371912; doi:10.3389/fpls.2021.710598)
Supplement: Supplementary file 1 [file Data_Sheet_1.docx]

**Supplementary Material**

**Metabolomic analysis provides new insight into tolerance of Huanglongbing in citrus**

**Joon Hyuk Suh^1^, Xixuan Tang^1^, Yi Zhang^1^, Frederick G. Gmitter Jr.^1^ & Yu Wang^1,^***

*^1^Citrus Research and Education Center, University of Florida, Lake Alfred, FL, United States*

***Correspondence**: Tel: +1-863-956-8673 E-mail: yu.wang@ufl.edu

**Supplementary Figure 1.** Morphological changes in HLB-tolerant and -sensitive cultivars after infection. (A) HLB-tolerant (Sugar Belle^®^) and (B) HLB-sensitive (Murcott) cultivars.


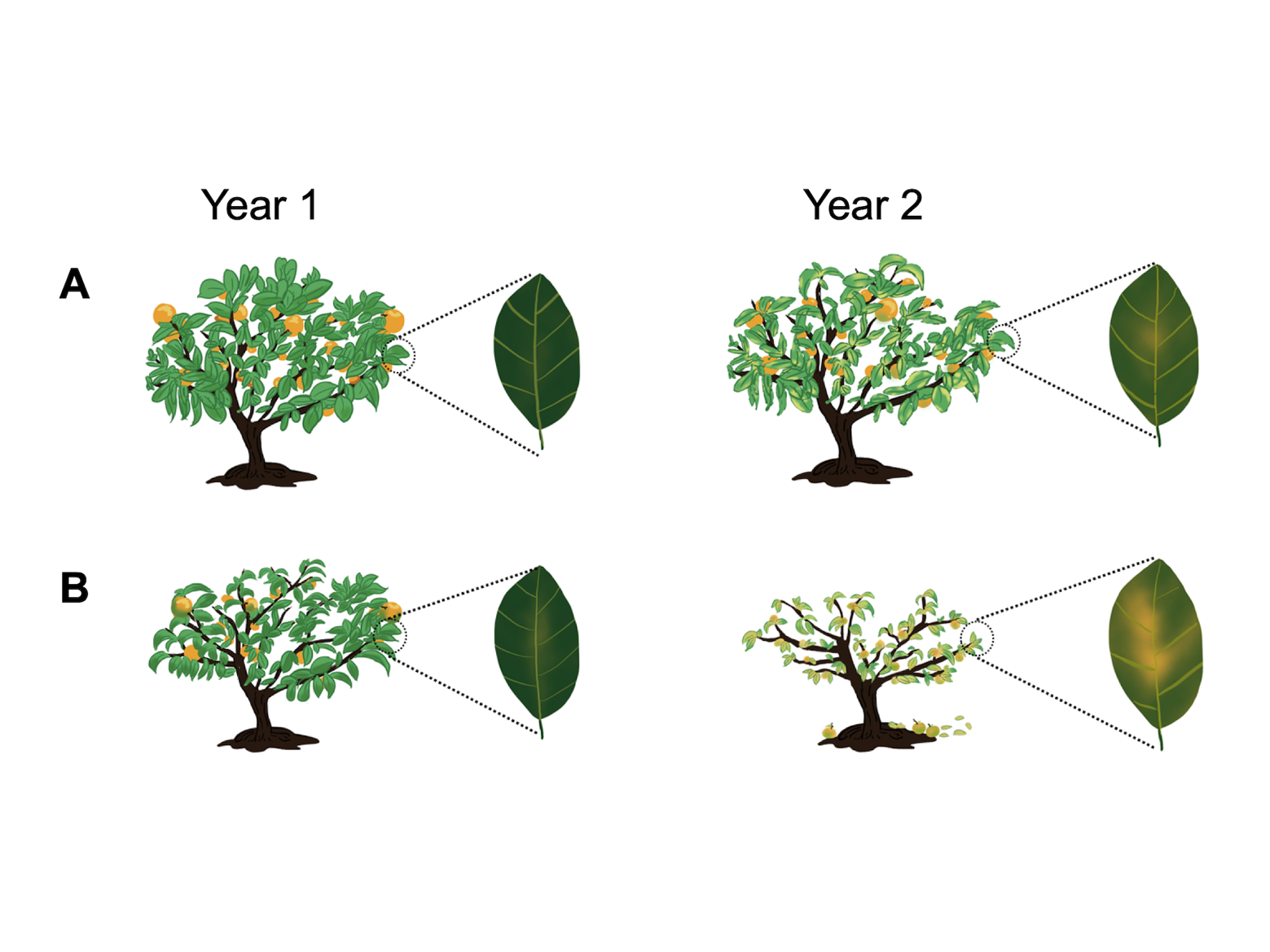


**Supplementary Figure 2.** PCA score scatter plots of (A) healthy and (B) infected groups: HLB-tolerant samples (green) and HLB-sensitive samples (red) (n = 9).

**
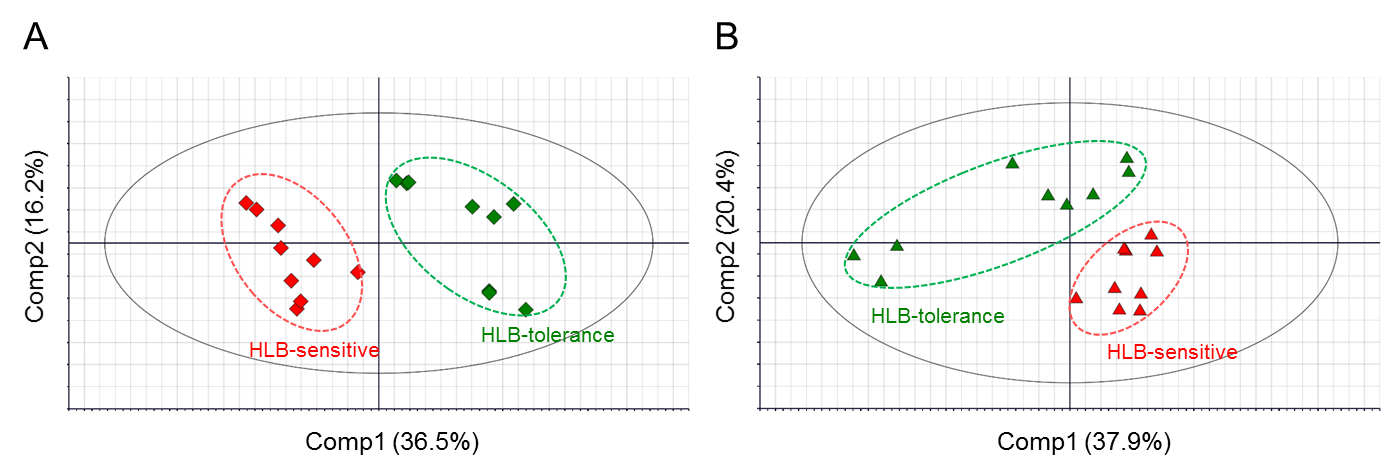
**

**Supplementary Figure 3.** Integrated results of marker compounds (criteria: VIP score > 1.0, p-value < 0.05 and |fold change| > 1.5)) based on healthy and infected HLB-tolerant and HLB-sensitive group comparisons (n = 9). Blue and yellow rounded rectangles indicate metabolites selected from healthy and infected group comparisons, respectively (see Supplementary Table 2 and 3). The overlapped region (green) indicates metabolites selected from both results. Metabolites with red color denote they are always increased in the HLB-tolerant group (behavior 1) regardless of healthy/infected status. Metabolites with blue color denote they are always decreased in the HLB-tolerant group (behavior 2) regardless of healthy/infected status. The rest of metabolites (black color) denote they show irregular response patterns between groups (behavior 3 and 4).

**
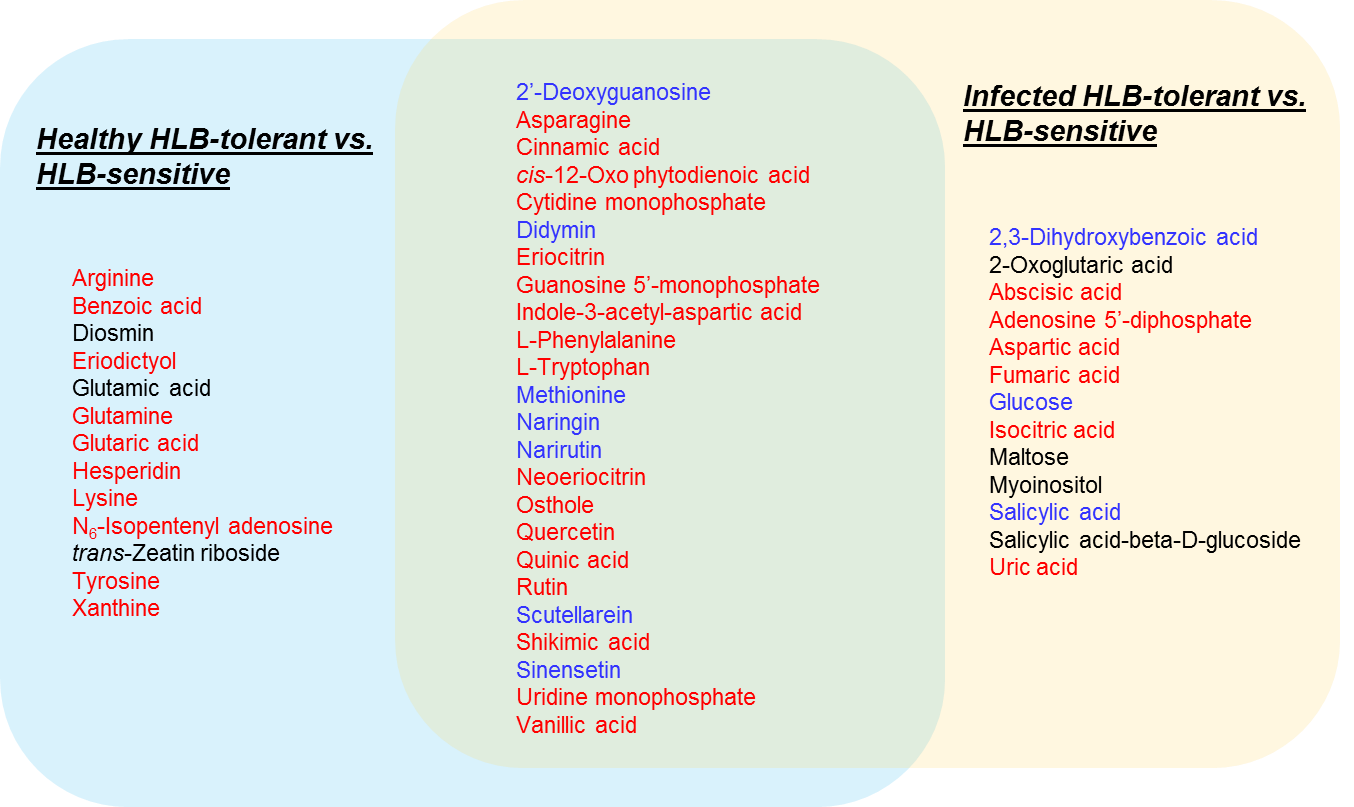
**

**Supplementary Figure 4.** A hierarchical clustering heatmap (n = 9) of metabolite markers (VIP score > 1.0, p-value < 0.05 and |fold change| > 1.5) showing constant metabolic behaviors (behavior 1 and 2). Behavior 1 indicates metabolic responses (average) always increased in the HLB-tolerant group. Behavior 2 indicates metabolic responses (average) always decreased in the HLB-tolerant group. The color depth of heatmap represents the degree of metabolic responses: red color denotes increased responses and blue color decreased responses.


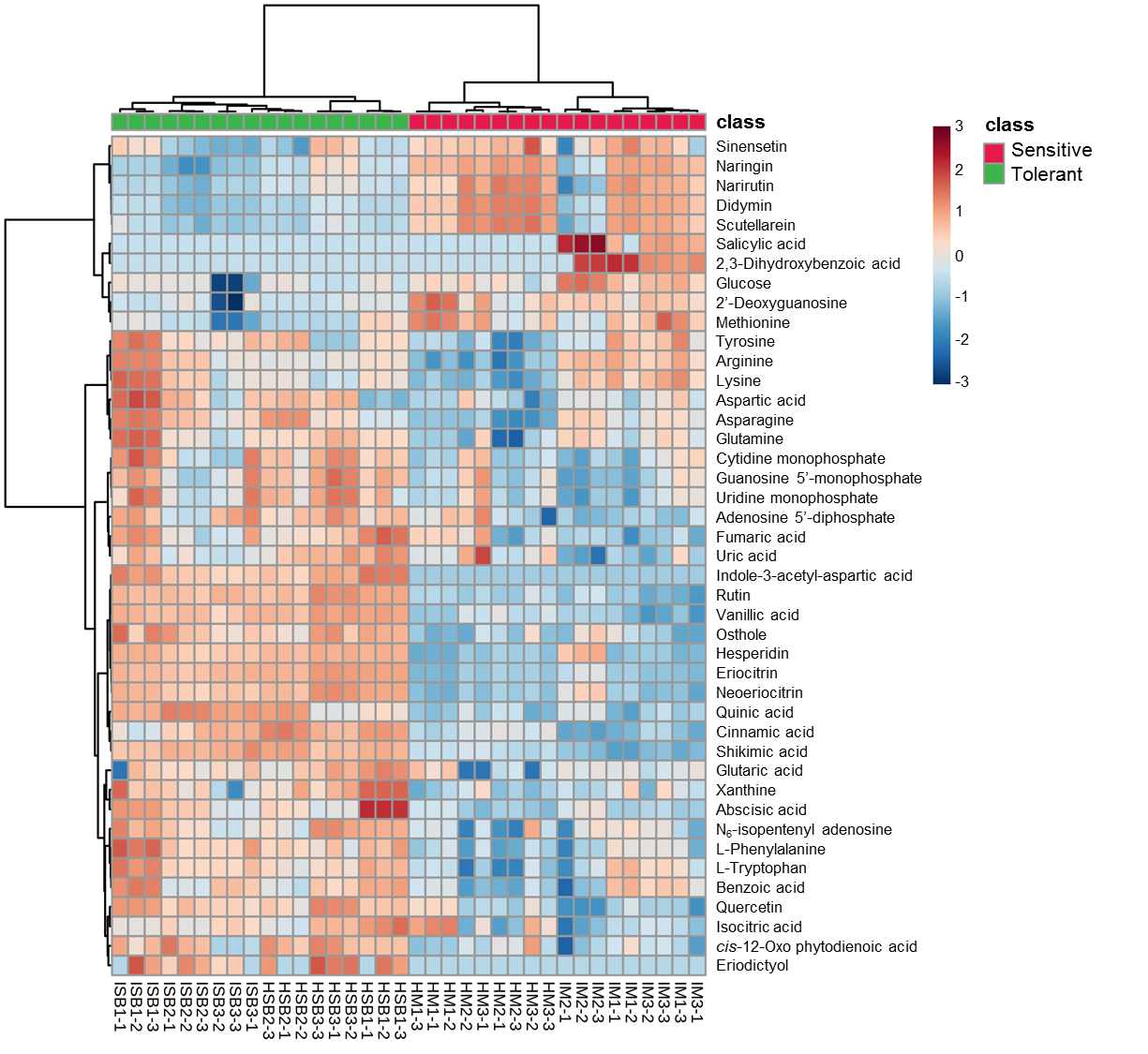


**Supplementary Table 1.** Information of 178 target metabolites, their optimum analytical conditions and identification methods.

| **Compound class** | **Compound name** | **Polarity** | **Transition** | **Collision energy** | **RF lens** | **Retention time (min)** | **Identification^a^** | | |
| --- | --- | --- | --- | --- | --- | --- | --- | --- | --- |
|  |  |  |  |  |  |  | **Column^b^** | **Level^c^** | **Reference**  **standard** |
| Internal standards | D-Fructose-^13^C_6_ | negative | 185.1/92.1 | 10 | 30 | 3.7 | HILIC | 1 | ○ |
|  | Citric-2,2,4,4-d_4_ acid | negative | 195.1/113.1 | 12 | 44 | 3.8 | C-30 | 1 | ○ |
|  | Guanine-4,5-^13^C2,7-^15^N | positive | 155/138.1 | 20 | 65 | 4.3 | HILIC | 1 | ○ |
|  | D-Sorbitol-^13^C_6_ | negative | 187.1/92.1 | 14 | 50 | 4.5 | HILIC | 1 | ○ |
|  | L-Aspartic acid-2,3,3-d_3_ | positive | 137.3/91.1 | 10 | 30 | 9.1 | HILIC | 1 | ○ |
|  | Hippuric acid-d_5_ | positive | 185/110.1 | 14 | 30 | 12.8 | C-30 | 1 | ○ |
|  | Salicylic acid-d_6_ | negative | 141.3/97.1 | 17 | 45 | 18.3 | C-30 | 1 | ○ |
|  | Apigenin-d_5_ | positive | 276.1/155 | 30 | 118 | 24.6 | C-30 | 1 | ○ |
|  |  |  |  |  |  |  |  |  |  |
| Sugars | Glycerol | negative | 91.2/59.1 | 10 | 30 | 1.6 | HILIC-1 | 1 | ○ |
|  | Rhamnose | negative | 209.0/163.1 | 10 | 34 | 2.8 | HILIC-1 | 1 | ○ |
|  | Erythritol | negative | 121.1/89.1 | 10 | 39 | 3.0 | HILIC-1 | 1 | ○ |
|  | Xylose | negative | 149/89 | 10 | 34 | 3.4 | HILIC-1 | 1 | ○ |
|  | Arabinose | negative | 149/89 | 10 | 30 | 3.5 | HILIC-1 | 1 | ○ |
|  | Arabitol | negative | 151/89.1 | 10 | 40 | 3.6 | HILIC-1 | 1 | ○ |
|  | Xylitol | negative | 151/89.1 | 10 | 43 | 3.7 | HILIC-1 | 1 | ○ |
|  | Fructose | negative | 179.1/89.1 | 10 | 30 | 3.7 | HILIC-1 | 1 | ○ |
|  | Mannitol | negative | 181/101 | 14 | 42 | 4.4 | HILIC-1 | 1 | ○ |
|  | Sorbitol | negative | 181.1/89.1 | 14 | 50 | 4.5 | HILIC-1 | 1 | ○ |
|  | Glucose | negative | 179.1/89.1 | 10 | 30 | 4.6 | HILIC-1 | 1 | ○ |
|  | Sucrose | negative | 341.1/179.1 | 14 | 66 | 6.4 | HILIC-1 | 1 | ○ |
|  | Myoinositol | negative | 179/87.1 | 17 | 53 | 6.8 | HILIC-1 | 1 | ○ |
|  | Maltose | negative | 341.1/161 | 10 | 38 | 7.0 | HILIC-1 | 1 | ○ |
|  | Melibiose | negative | 341.1/179.1 | 10 | 30 | 7.8 | HILIC-1 | 1 | ○ |
|  | Raffinose | negative | 503.2/179.1 | 21 | 91 | 8.7 | HILIC-1 | 1 | ○ |
|  |  |  |  |  |  |  |  |  |  |
| Amino acids | Leucine | positive | 132.1/86.2 | 10 | 30 | 3.8 | HILIC-1 | 1 | ○ |
|  | Isoleucine | positive | 132.1/69.1 | 17 | 33 | 4.2 | HILIC-1 | 1 | ○ |
|  | Methionine | positive | 150.1/104.1 | 10 | 38 | 4.5 | HILIC-1 | 1 | ○ |
|  | Tyrosine | positive | 182/136.1 | 14 | 36 | 4.9 | HILIC-1 | 1 | ○ |
|  | Valine | positive | 118.2/72.1 | 10 | 30 | 5.2 | HILIC-1 | 1 | ○ |
|  | Theanine | positive | 175/158.1 | 10 | 40 | 5.5 | HILIC-1 | 1 | ○ |
|  | Proline | positive | 116.2/70.1 | 16 | 34 | 5.7 | HILIC-1 | 1 | ○ |
|  | Cysteine | positive | 122/59.1 | 23 | 30 | 6.0 | HILIC-1 | 1 | ○ |
|  | Alanine | positive | 90.1/44.2 | 10 | 30 | 6.9 | HILIC-1 | 1 | ○ |
|  | Threonine | positive | 120.1/74.1 | 10 | 30 | 6.9 | HILIC-1 | 1 | ○ |
|  | Glycine | positive | 76/76 | 10 | 30 | 7.5 | HILIC-1 | 1 | ○ |
|  | Serine | positive | 106/60.1 | 10 | 30 | 7.6 | HILIC-1 | 1 | ○ |
|  | Glutamine | positive | 147.2/84.1 | 17 | 31 | 7.6 | HILIC-1 | 1 | ○ |
|  | Asparagine | positive | 133.2/74.1 | 16 | 34 | 7.8 | HILIC-1 | 1 | ○ |
|  | L-Phenylalanine | positive | 166.1/120.1 | 14 | 37 | 8.3 | C-30 | 1 | ○ |
|  | Histidine | positive | 156.1/110.1 | 15 | 47 | 8.9 | HILIC-1 | 1 | ○ |
|  | Aspartic acid | positive | 134/74.1 | 15 | 30 | 9.1 | HILIC-1 | 1 | ○ |
|  | Glutamic acid | positive | 148/84.1 | 16 | 30 | 9.2 | HILIC-1 | 1 | ○ |
|  | L-Tryptophan | positive | 205.1/188.1 | 10 | 37 | 11.8 | C-30 | 1 | ○ |
|  | Arginine | positive | 175/70.2 | 23 | 55 | 12.4 | HILIC-1 | 1 | ○ |
|  | Lysine | positive | 147.1/84.2 | 17 | 38 | 13.2 | HILIC-1 | 1 | ○ |
|  |  |  |  |  |  |  |  |  |  |
| Organic acids | Cinnamic acid | negative | 147.1/103.1 | 10 | 46 | 2.1 | HILIC-1 | 1 | ○ |
|  | Oxalic acid | negative | 89/61.1 | 10 | 30 | 2.3 | C-30 | 1 | ○ |
|  | Tartaric acid | negative | 149/87.1 | 13 | 48 | 2.4 | C-30 | 1 | ○ |
|  | Maleic acid | negative | 115.1/71.1 | 10 | 34 | 2.5 | HILIC-1 | 1 | ○ |
|  | Malic acid | negative | 133.1/115.1 | 10 | 42 | 2.5 | C-30 | 1 | ○ |
|  | Pyruvic acid | negative | 87.2/43.2 | 10 | 41 | 2.5 | C-30 | 1 | ○ |
|  | Oxaloacetic acid | negative | 131.1/87.2 | 10 | 49 | 2.5 | C-30 | 1 | ○ |
|  | Ferulic acid | negative | 193/134.1 | 16 | 54 | 2.6 | HILIC-1 | 1 | ○ |
|  | Ascorbic acid | negative | 175.1/115.1 | 10 | 56 | 2.6 | C-30 | 1 | ○ |
|  | Isocitric acid | negative | 191.1/111.1 | 14 | 48 | 2.6 | C-30 | 1 | ○ |
|  | Coumaric acid | negative | 163.1/119.1 | 16 | 50 | 2.7 | HILIC-1 | 1 | ○ |
|  | Vanillic acid | negative | 167.1/152.1 | 14 | 51 | 3.1 | HILIC-1 | 1 | ○ |
|  | Citric acid | negative | 191.1/111.1 | 10 | 45 | 3.8 | C-30 | 1 | ○ |
|  | Uric acid | negative | 167/124 | 14 | 65 | 4.8 | HILIC-1 | 1 | ○ |
|  | Quinic acid | negative | 191.1/85.1 | 21 | 67 | 6.6 | HILIC-1 | 1 | ○ |
|  | Shikimic acid | negative | 173.1/93.1 | 14 | 47 | 7.4 | HILIC-1 | 1 | ○ |
|  | Malonic acid | negative | 103.1/59.1 | 10 | 30 | 8.2 | HILIC-1 | 1 | ○ |
|  | 2-Oxoglutaric acid | negative | 145/101.1 | 10 | 33 | 8.5 | HILIC-1 | 1 | ○ |
|  | Gallic acid | negative | 169.1/125.1 | 15 | 59 | 8.7 | C-30 | 1 | ○ |
|  | Succinic acid | negative | 117.1/73.1 | 10 | 39 | 9.3 | HILIC-1 | 1 | ○ |
|  | Fumaric acid | negative | 115.3/71.1 | 11 | 40 | 9.4 | HILIC-1 | 1 | ○ |
|  | Glutaric acid | negative | 131.1/87.1 | 10 | 41 | 9.7 | HILIC-1 | 1 | ○ |
|  |  |  |  |  |  |  |  |  |  |
| Nucleosides | Thymidine | negative | 241.1/151.1 | 10 | 55 | 1.8 | HILIC-2 | 1 | ○ |
|  | Thymine | negative | 125/42.2 | 15 | 47 | 1.9 | HILIC-2 | 1 | ○ |
|  | 2'-Deoxyadenosine | positive | 252.1/136.1 | 16 | 44 | 2.2 | HILIC-2 | 1 | ○ |
|  | 2'-Deoxyuridine | negative | 227.1/184 | 10 | 50 | 2.2 | HILIC-2 | 1 | ○ |
|  | Uracil | negative | 111/42.2 | 15 | 47 | 2.3 | HILIC-2 | 1 | ○ |
|  | 5-Methyluridine | negative | 257.1/167.1 | 10 | 61 | 2.4 | HILIC-2 | 1 | ○ |
|  | Adenine | positive | 136.3/119.1 | 23 | 66 | 2.7 | HILIC-2 | 1 | ○ |
|  | Adenosine | positive | 268.1/136.1 | 19 | 53 | 2.7 | HILIC-2 | 1 | ○ |
|  | 2'-Deoxyinosine | negative | 251.1/135.1 | 21 | 73 | 3.2 | HILIC-2 | 1 | ○ |
|  | Uridine | negative | 243.1/200.1 | 10 | 52 | 3.3 | HILIC-2 | 1 | ○ |
|  | Hypoxanthine | negative | 135.2/92.1 | 17 | 60 | 3.4 | HILIC-2 | 1 | ○ |
|  | 2'-Deoxycytidine | positive | 228.1/112.1 | 10 | 31 | 3.7 | HILIC-2 | 1 | ○ |
|  | Cytosine | positive | 112.3/95.1 | 19 | 61 | 3.8 | HILIC-2 | 1 | ○ |
|  | 5-Methylcytidine | positive | 258.1/126.1 | 15 | 41 | 3.8 | HILIC-2 | 1 | ○ |
|  | 2'-Deoxyguanosine | negative | 266.1/150.1 | 19 | 74 | 4.0 | HILIC-2 | 1 | ○ |
|  | Inosine | negative | 267.2/135 | 22 | 72 | 4.0 | HILIC-2 | 1 | ○ |
|  | Xanthine | negative | 151/108.1 | 17 | 67 | 4.1 | HILIC-2 | 1 | ○ |
|  | Cytidine | positive | 244.1/112.1 | 13 | 35 | 4.2 | HILIC-2 | 1 | ○ |
|  | Guanine | positive | 152.3/135.1 | 19 | 62 | 4.3 | HILIC-2 | 1 | ○ |
|  | Guanosine | negative | 282.1/150.1 | 18 | 74 | 4.4 | HILIC-2 | 1 | ○ |
|  | Xanthosine | negative | 283.2/151.1 | 19 | 82 | 5.0 | HILIC-2 | 1 | ○ |
|  |  |  |  |  |  |  |  |  |  |
| Nucleotides | Adenosine 3',5'-cyclic monophosphate | positive | 330.1/136.1 | 25 | 76 | 4.5 | HILIC-2 | 1 | ○ |
|  | 2'-Deoxyadenosine 5'-monophosphate | positive | 332.1/136.1 | 17 | 58 | 4.9 | HILIC-2 | 1 | ○ |
|  | Thymidine 5′-monophosphate | negative | 321/195.1 | 17 | 70 | 4.9 | HILIC-2 | 1 | ○ |
|  | Guanosine 3′,5′-cyclic monophosphate | positive | 346/152.1 | 21 | 64 | 5.1 | HILIC-2 | 1 | ○ |
|  | 2'-Deoxyuridine 5'-monophosphate | negative | 307/195.1 | 16 | 63 | 5.1 | HILIC-2 | 1 | ○ |
|  | Adenosine 5'-monophosphate | positive | 348.1/136.1 | 20 | 64 | 5.1 | HILIC-2 | 1 | ○ |
|  | 2'-Deoxyinosine-5'-monophosphate | negative | 331/195 | 16 | 67 | 5.2 | HILIC-2 | 1 | ○ |
|  | 2'-Deoxycytidine-5'-monophosphate | positive | 308.1/112.1 | 10 | 38 | 5.3 | HILIC-2 | 1 | ○ |
|  | 2'-Deoxyguanosine 5'-monophosphate | positive | 348.1/152.1 | 13 | 47 | 5.4 | HILIC-2 | 1 | ○ |
|  | 2'-Deoxyadenosine-5'-diphosphate | positive | 412.1/136.1 | 19 | 64 | 5.4 | HILIC-2 | 1 | ○ |
|  | 2'-Deoxythymidine-5'-diphosphate | negative | 401/275 | 19 | 78 | 5.4 | HILIC-2 | 1 | ○ |
|  | Inosine 5'-monophosphate | positive | 349/137.1 | 15 | 48 | 5.4 | HILIC-2 | 1 | ○ |
|  | Uridine 5'-monophosphate | negative | 323/211.1 | 15 | 71 | 5.4 | HILIC-2 | 1 | ○ |
|  | Adenosine 5'-diphosphate | positive | 428/136.1 | 25 | 72 | 5.5 | HILIC-2 | 1 | ○ |
|  | Cytidine 5'-monophosphate | positive | 324.1/112.1 | 16 | 47 | 5.5 | HILIC-2 | 1 | ○ |
|  | 2′-Deoxycytidine 5′-diphosphate | positive | 388.1/112.1 | 16 | 51 | 5.7 | HILIC-2 | 1 | ○ |
|  | 2′-Deoxyadenosine 5′-triphosphate | positive | 492/136.1 | 21 | 69 | 5.7 | HILIC-2 | 1 | ○ |
|  | Guanosine 5'-monophosphate | positive | 364.1/152.1 | 20 | 64 | 5.7 | HILIC-2 | 1 | ○ |
|  | Uridine-5'-diphosphate | positive | 405/97.1 | 17 | 58 | 5.8 | HILIC-2 | 1 | ○ |
|  | Cytidine-5'-diphosphate | positive | 404/112.1 | 19 | 56 | 5.9 | HILIC-2 | 1 | ○ |
|  | 2′-Deoxyguanosine 5′-diphosphate | positive | 428/152.1 | 18 | 48 | 5.9 | HILIC-2 | 1 | ○ |
|  | Adenosine 5′-triphosphate | positive | 508/410.1 | 17 | 79 | 5.9 | HILIC-2 | 1 | ○ |
|  | Thymidine 5′-triphosphate | negative | 481/383.1 | 21 | 86 | 5.9 | HILIC-2 | 1 | ○ |
|  | 2'-Deoxyuridine-5'-triphosphate | negative | 467/369 | 20 | 86 | 6.0 | HILIC-2 | 1 | ○ |
|  | Guanosine 5′-diphosphate | positive | 444/152.1 | 20 | 68 | 6.1 | HILIC-2 | 1 | ○ |
|  | 2′-Deoxycytidine 5′-triphosphate | positive | 468/112.1 | 18 | 58 | 6.1 | HILIC-2 | 1 | ○ |
|  | Xanthosine 5'-monophosphate | negative | 363/211.1 | 18 | 74 | 6.1 | HILIC-2 | 1 | ○ |
|  | Inosine 5′-triphosphate | positive | 509/137.1 | 22 | 74 | 6.2 | HILIC-2 | 1 | ○ |
|  | Uridine-5'-triphosphate | negative | 483/385 | 19 | 89 | 6.2 | HILIC-2 | 1 | ○ |
|  | 2′-Deoxyguanosine 5′-triphosphate | positive | 508/152.1 | 21 | 66 | 6.3 | HILIC-2 | 1 | ○ |
|  | Cytidine-5'-triphosphate | positive | 484/112.1 | 22 | 69 | 6.4 | HILIC-2 | 1 | ○ |
|  | Guanosine-5'-Triphosphate | positive | 524/152.1 | 25 | 76 | 6.5 | HILIC-2 | 1 | ○ |
|  |  |  |  |  |  |  |  |  |  |
| Flavonoids | Eriocitrin | negative | 595.2/287 | 23 | 91 | 16.4 | C-30 | 1 | ○ |
|  | Neoeriocitrin | negative | 595.2/287 | 23 | 91 | 16.8 | C-30 | 1 | ○ |
|  | Rutin | negative | 609.2/300 | 10 | 109 | 17.0 | C-30 | 1 | ○ |
|  | Narirutin | negative | 579.2/295.1 | 25 | 95 | 17.6 | C-30 | 1 | ○ |
|  | Taxifolin | negative | 303/285.1 | 10 | 61 | 17.8 | C-30 | 1 | ○ |
|  | Naringin | negative | 579.2/271.1 | 32 | 116 | 18.1 | C-30 | 1 | ○ |
|  | Hesperidin | negative | 609.2/325.1 | 27 | 90 | 18.3 | C-30 | 1 | ○ |
|  | Rhoifolin | positive | 579.2/271.1 | 25 | 92 | 18.3 | C-30 | 1 | ○ |
|  | Diosmin | positive | 609.2/301 | 23 | 93 | 18.4 | C-30 | 1 | ○ |
|  | Neohesperidin | negative | 609.2/301 | 32 | 95 | 18.8 | C-30 | 1 | ○ |
|  | Neodiosmin | positive | 609.2/301 | 23 | 93 | 18.8 | C-30 | 1 | ○ |
|  | Scutellarein | positive | 287.1/123.1 | 33 | 123 | 20.9 | C-30 | 1 | ○ |
|  | Didymin | negative | 593.2/309.1 | 27 | 95 | 21.1 | C-30 | 1 | ○ |
|  | Poncirin | negative | 593.2/285.1 | 30 | 105 | 21.5 | C-30 | 1 | ○ |
|  | Eriodictyol | positive | 289.1/153.1 | 24 | 75 | 21.6 | C-30 | 1 | ○ |
|  | Quercetin | negative | 301/151 | 21 | 77 | 22.6 | C-30 | 1 | ○ |
|  | Luteolin | negative | 285.1/133.1 | 35 | 97 | 22.8 | C-30 | 1 | ○ |
|  | Naringenin | positive | 273.1/153.1 | 23 | 73 | 23.8 | C-30 | 1 | ○ |
|  | Hesperetin | positive | 303.1/171.1 | 18 | 74 | 24.5 | C-30 | 1 | ○ |
|  | Apigenin | positive | 271.1/153.1 | 30 | 111 | 24.6 | C-30 | 1 | ○ |
|  | Diosmetin | positive | 301.1/286 | 12 | 101 | 25.4 | C-30 | 1 | ○ |
|  | Limonin | positive | 471.2/425.3 | 19 | 81 | 27.0 | C-30 | 1 | ○ |
|  | Sinensetin | positive | 373.2/343 | 14 | 95 | 27.1 | C-30 | 1 | ○ |
|  | 5,6,7,3’,4’,5’-Hexamethoxyflavone | positive | 403/359.1 | 27 | 97 | 28.0 | C-30 | 1 | ○ |
|  | Nobiletin | positive | 403/359.1 | 27 | 97 | 28.5 | C-30 | 1 | ○ |
|  | Nomilin | positive | 515.3/411.3 | 15 | 78 | 28.5 | C-30 | 1 | ○ |
|  | Isosakuranetin | positive | 287.1/153.1 | 23 | 74 | 28.6 | C-30 | 1 | ○ |
|  | Tangeretin | positive | 373.2/312.1 | 26 | 95 | 29.7 | C-30 | 1 | ○ |
|  | Osthole | positive | 245.1/189.1 | 14 | 58 | 30.8 | C-30 | 1 | ○ |
|  |  |  |  |  |  |  |  |  |  |
| Plant hormones | *trans*-Zeatin | positive | 220.1/136.1 | 19 | 59 | 10.7 | C-30 | 1 | ○ |
|  | *cis*-Zeatin | positive | 220.2/136.1 | 17 | 56 | 10.9 | C-30 | 1 | ○ |
|  | Salicylic acid-beta-D-glucoside | negative | 299.1/137.1 | 15 | 68 | 12.0 | C-30 | 1 | ○ |
|  | *trans*-Zeatin riboside | positive | 352.2/220.1 | 19 | 71 | 12.5 | C-30 | 1 | ○ |
|  | Dihydrozeatin riboside | positive | 354.2/222.1 | 20 | 72 | 12.5 | C-30 | 1 | ○ |
|  | 2,3-Dihydroxybenzoic acid | negative | 153/109.1 | 16 | 51 | 14.1 | C-30 | 1 | ○ |
|  | N_6_-(Δ2-Isopentenyl)adenine | positive | 204.1/136.1 | 16 | 52 | 14.5 | C-30 | 1 | ○ |
|  | Oxindole-3-acetic acid | negative | 190/146.1 | 14 | 53 | 14.9 | C-30 | 1 | ○ |
|  | Indole-3-acetamide | positive | 175.1/130.1 | 17 | 41 | 15.1 | C-30 | 1 | ○ |
|  | Indole-3-acetyl-L-aspartic acid | positive | 291.1/130.1 | 23 | 48 | 15.6 | C-30 | 1 | ○ |
|  | Indole-3-acetyl glycine | positive | 233.1/130.1 | 21 | 44 | 15.9 | C-30 | 1 | ○ |
|  | N_6_-Isopentenyl adenosine | positive | 336.2/204.1 | 18 | 69 | 16.2 | C-30 | 1 | ○ |
|  | Indole-3-acetyl-L-glutamic acid | positive | 305.1/130.1 | 24 | 56 | 16.3 | C-30 | 1 | ○ |
|  | Gibberellic acid | negative | 345.2/239.1 | 14 | 69 | 16.3 | C-30 | 1 | ○ |
|  | Benzoic acid | negative | 121.1/77.1 | 10 | 40 | 17.5 | C-30 | 1 | ○ |
|  | Indole-3-carboxylic acid | negative | 160.2/116.1 | 16 | 48 | 17.7 | C-30 | 1 | ○ |
|  | Salicylic acid | negative | 137.3/93.1 | 17 | 50 | 18.4 | C-30 | 1 | ○ |
|  | Indole-3-acetic acid | positive | 176.1/130.1 | 17 | 45 | 19.1 | C-30 | 1 | ○ |
|  | Abscisic acid | negative | 263.1/153.1 | 10 | 48 | 20.2 | C-30 | 1 | ○ |
|  | Gibberellin A5 | negative | 329.1/285.2 | 17 | 73 | 20.7 | C-30 | 1 | ○ |
|  | 3-Indoleacetonitrile | positive | 157.3/130.1 | 13 | 60 | 22.6 | C-30 | 1 | ○ |
|  | Jasmonic acid | negative | 209/59.1 | 13 | 48 | 22.8 | C-30 | 1 | ○ |
|  | Indole-3-butyric acid | negative | 202.1/158.2 | 14 | 63 | 23.5 | C-30 | 1 | ○ |
|  | 9,10-Dihydrojasmonic acid | negative | 211.1/59.2 | 13 | 56 | 24.8 | C-30 | 1 | ○ |
|  | Gibberellin A7 | negative | 329.1/223.2 | 18 | 70 | 25.2 | C-30 | 1 | ○ |
|  | Gibberellin A4 | negative | 331.2/257.1 | 23 | 74 | 25.4 | C-30 | 1 | ○ |
|  | Jasmonic acid-isoleucine | negative | 322.2/130.2 | 21 | 79 | 25.6 | C-30 | 1 | ○ |
|  | Methyl salicylate | positive | 153.1/121.1 | 15 | 43 | 25.7 | C-30 | 1 | ○ |
|  | Dinor-12-oxo phytodienoic acid | positive | 265.2/247.2 | 12 | 58 | 28.4 | C-30 | 1 | ○ |
|  | Epibrassinolide | positive | 481.4/445.4 | 10 | 63 | 29.0 | C-30 | 1 | ○ |
|  | *cis*-12-Oxo phytodienoic acid | positive | 293.2/275.2 | 12 | 57 | 30.8 | C-30 | 1 | ○ |
|  |  |  |  |  |  |  |  |  |  |
| Others | Trehalose | negative | 341.1/179.1 | 14 | 79 | 5.1 | HILIC-2 | 1 | ○ |
|  | Fructose-6-phosphate | negative | 259/97.1 | 15 | 47 | 5.5 | HILIC-2 | 1 | ○ |
|  | Trehalose-6-phosphate | negative | 421.1/241.1 | 26 | 101 | 5.7 | HILIC-2 | 1 | ○ |
|  | Glucose-6-phosphate | negative | 259/97.1 | 15 | 49 | 5.8 | HILIC-2 | 1 | ○ |
|  | 3-Phosphoglycerate | negative | 185/97.1 | 15 | 50 | 6.0 | HILIC-2 | 1 | ○ |
|  | Phosphoenolpyruvate | negative | 167/79.1 | 13 | 35 | 6.1 | HILIC-2 | 1 | ○ |

^a^ Identification confirmed by spectral and chromatographic properties by comparison with authentic standards.

^b^ Metabolites including internal standards were analyzed by different columns: HILIC (HILIC-1 and HILIC-2) and C-30.

^c^ Level of metabolite identification as follows: (1) Identified; (2) Putatively annotated; (3) Putatively characterized metabolite classes; (4) Unknown (Bino *et al.*, 2004, Sumner *et al.*, 2007).

**Supplementary Table 2.** Metabolite markers selected from comparison between healthy HLB-tolerant and HLB-sensitive groups (n = 9) (criteria: VIP score > 1.0, p-value < 0.05 and |fold change| > 1.5).

| **Metabolite** | **VIP score** | **P-value** | **Fold change (T/S)^a^** |
| --- | --- | --- | --- |
| Hesperidin | 1.66 | 2.3E-12 | 28.5 |
| Vanillic acid | 1.63 | 3.0E-10 | 3.1 |
| Shikimic acid | 1.59 | 7.5E-08 | 9.6 |
| Arginine | 1.59 | 1.2E-08 | 4.7 |
| Cinnamic acid | 1.56 | 4.5E-07 | 4.6 |
| Eriocitrin | 1.54 | 2.4E-07 | 163.4 |
| Naringin | 1.53 | 5.4E-07 | -3.7 |
| Rutin | 1.49 | 1.9E-06 | 11.8 |
| Neoeriocitrin | 1.46 | 4.7E-06 | 55.4 |
| Lysine | 1.46 | 6.2E-06 | 2.4 |
| Indole-3-acetyl-aspartic acid | 1.41 | 2.3E-05 | Invalid^#^ |
| L-Tryptophan | 1.40 | 2.3E-05 | 2.4 |
| L-Phenylalanine | 1.40 | 2.5E-05 | 2.6 |
| Glutamic acid | 1.40 | 3.4E-05 | 8.5 |
| Osthole | 1.40 | 2.8E-05 | 3.9 |
| Glutamine | 1.40 | 3.2E-05 | 2.3 |
| Didymin | 1.34 | 1.0E-04 | -24.1 |
| Scutellarein | 1.32 | 1.4E-04 | -15.2 |
| Diosmin | 1.31 | 2.0E-04 | 5.9 |
| Asparagine | 1.30 | 7.4E-04 | 4.2 |
| Narirutin | 1.29 | 2.5E-04 | -4.8 |
| Xanthine | 1.27 | 3.8E-04 | 4.3 |
| Benzoic acid | 1.23 | 6.9E-04 | 2.5 |
| *trans*-Zeatin riboside | 1.21 | 9.6E-04 | 2.1 |
| *cis*-12-Oxo phytodienoic acid | 1.20 | 1.2E-03 | 3.2 |
| N_6_-isopentenyl adenosine | 1.17 | 1.7E-03 | 2.2 |
| Tyrosine | 1.17 | 2.6E-03 | 1.7 |
| Quinic acid | 1.17 | 6.4E-03 | 7.0 |
| Cytidine monophosphate | 1.15 | 2.3E-03 | 2.8 |
| Methionine | 1.15 | 3.9E-03 | -1.7 |
| Glutaric acid | 1.14 | 2.5E-03 | 3.1 |
| 2’-Deoxyguanosine | 1.11 | 4.4E-03 | -2.0 |
| Eriodictyol | 1.10 | 4.1E-03 | Invalid^#^ |
| Guanosine 5’-monophosphate | 1.08 | 7.4E-03 | 2.5 |
| Uridine monophosphate | 1.03 | 1.0E-02 | 2.8 |
| Sinensetin | 1.02 | 1.1E-02 | -1.7 |
| Quercetin | 1.00 | 1.1E-02 | 7.9 |

^a^Tolerant/sensitive

^#^Only found in HLB-tolerant group

**Supplementary Table 3.** Metabolite markers selected from comparison between infected HLB-tolerant and HLB-sensitive groups (n = 9) (criteria: VIP score > 1.0, p-value < 0.05 and |fold change| > 1.5).

| **Metabolite** | **VIP score** | **P-value** | **Fold change (T/S)^a^** |
| --- | --- | --- | --- |
| Rutin | 1.83 | 8.6E-12 | 10.9 |
| Vanillic acid | 1.80 | 5.6E-11 | 3.9 |
| Eriocitrin | 1.75 | 7.3E-09 | 29.7 |
| Quinic acid | 1.75 | 1.8E-07 | 11.4 |
| Maltose | 1.65 | 1.3E-06 | Invalid* |
| Shikimic acid | 1.63 | 4.1E-06 | 32.5 |
| Isocitric acid | 1.59 | 1.8E-05 | 2.5 |
| Cinnamic acid | 1.58 | 6.3E-05 | 5.9 |
| 2’-Deoxyguanosine | 1.53 | 8.0E-05 | -1.9 |
| Glucose | 1.51 | 1.3E-04 | -2.3 |
| Indole-3-acetyl-aspartic acid | 1.49 | 6.4E-05 | Invalid^#^ |
| Neoeriocitrin | 1.48 | 7.8E-05 | 4.8 |
| 2,3-Dihydroxybenzoic acid | 1.46 | 1.6E-04 | Invalid* |
| Naringin | 1.36 | 6.7E-04 | -8.2 |
| Salicylic acid beta-D-glucoside | 1.35 | 1.9E-03 | -2.5 |
| L-Phenylalanine | 1.34 | 9.4E-04 | 2.8 |
| Fumaric acid | 1.34 | 1.2E-03 | 1.8 |
| Quercetin | 1.31 | 1.4E-03 | 36.9 |
| Osthole | 1.29 | 1.4E-03 | 3.2 |
| Didymin | 1.27 | 2.0E-03 | -17.8 |
| Methionine | 1.25 | 3.9E-03 | -1.9 |
| Abscisic acid | 1.25 | 3.0E-03 | 3.9 |
| Adenosine 5’-diphosphate | 1.24 | 2.9E-03 | 11.5 |
| Scutellarein | 1.23 | 3.2E-03 | -9.9 |
| Myoinositol | 1.20 | 4.8E-03 | 1.5 |
| Narirutin | 1.19 | 4.6E-03 | -5.9 |
| Aspartic acid | 1.10 | 1.6E-02 | 1.8 |
| Uric acid | 1.09 | 1.4E-02 | 2.5 |
| *cis*-12-Oxo phytodienoic acid | 1.08 | 1.6E-02 | 3.8 |
| Asparagine | 1.07 | 2.0E-02 | 1.7 |
| Guanosine 5’-monophosphate | 1.06 | 2.4E-02 | 3.0 |
| Sinensetin | 1.05 | 2.4E-02 | -1.8 |
| Salicylic acid | 1.04 | 2.2E-02 | Invalid* |
| Cytidine monophosphate | 1.04 | 3.3E-02 | 3.5 |
| 2-Oxoglutaric acid | 1.03 | 1.7E-02 | -4.2 |
| L-Tryptophan | 1.03 | 2.2E-02 | 1.6 |
| Uridine monophosphate | 1.02 | 3.3E-02 | 4.2 |

^a^Tolerant/sensitive

^#^Only found in HLB-tolerant group

*Only found in HLB-sensitive group
